# Glutamine synthetase in *Medicago truncatula*, unveiling new secrets of a very old enzyme

**DOI:** 10.3389/fpls.2015.00578

**Published:** 2015-07-27

**Authors:** Ana R. Seabra, Helena G. Carvalho

**Affiliations:** Laboratory of Molecular Biology of Nitrogen Assimilation, Instituto de Biologia Molecular e Celular, Universidade do Porto, Porto, Portugal

**Keywords:** glutamine synthetase, *Medicago truncatula*, nitrogen metabolism, post-translational regulation, seed metabolism

## Abstract

Glutamine synthetase (GS) catalyzes the first step at which nitrogen is brought into cellular metabolism and is also involved in the reassimilation of ammonium released by a number of metabolic pathways. Due to its unique position in plant nitrogen metabolism, GS plays essential roles in all aspects of plant development, from germination to senescence, and is a key component of nitrogen use efficiency (NUE) and plant yield. Understanding the mechanisms regulating GS activity is therefore of utmost importance and a great effort has been dedicated to understand how GS is regulated in different plant species. The present review summarizes exciting recent developments concerning the structure and regulation of GS isoenzymes, using the model legume *Medicago truncatula*. These include the understanding of the structural determinants of both the cytosolic and plastid located isoenzymes, the existence of a seed-specific GS gene unique to *M. truncatula* and closely related species and the discovery that GS isoenzymes are regulated by nitric oxide at the post-translational level. The data is discussed and integrated with the potential roles of the distinct GS isoenzymes within the whole plant context.

## Introduction

Glutamine synthetase (GS, E.C. 6.3.1.2) is a tightly controlled enzyme located at the core of nitrogen metabolism. GS catalyzes the first step in nitrogen assimilation, the ATP-dependent condensation of ammonium with glutamate. This reaction yields the readily metabolizable glutamine, which is used for the synthesis of all other essential nitrogenous compounds contained in the cells. Besides this key role in primary nitrogen assimilation, GS is crucial for the reassimilation of the NH_4_^+^ constantly released in large quantities via processes such as photorespiration, lignin biosynthesis, and protein catabolism (Lea and Miflin, 2010). The enzyme is thus essential for the initial incorporation of inorganic nitrogen into an organic composition but also fulfills a broad spectrum of functions beyond primary metabolism in nitrogen reassimilation and amino acid metabolism.

Being involved in all aspects of nitrogen metabolism, GS is a key component of nitrogen use efficiency (NUE) and plant yield, justifying the extensive amount of studies that have been dedicated to understand how GS is regulated and how it regulates nitrogen metabolism in plants (for recent reviews see, Bernard and Habash, 2009; Lea and Miflin, 2010; Thomsen et al., 2014). To achieve its multiple roles in plant metabolism, GS belongs to a small multigene family encoding 4–6 isoenzymes located in the cytosol (GS1) and in the plastids (GS2). The GS genes follow a complex pattern of expression, influenced by developmental and environmental cues, and their encoded enzymes participate in different metabolic processes. Some genes show an organ/tissue specific expression and others are ubiquitously expressed. GS2 is highly expressed in photosynthetic tissues and plays a major role in the reassimilation of NH_4_^+^ released during photorespiration and in the assimilation of NH_4_^+^ deriving from NO_3_^–^ reduction in plastids (Wallsgrove et al., 1987; Orea et al., 2002). GS1 is important for primary NH_4_^+^ assimilation in roots and for reassimilation of NH_4_^+^ generated by several different processes occurring in the cytosol, including amino-acid catabolism and the activities of phenylalanine ammonia lyase and asparaginase (Bernard and Habash, 2009; Lea and Miflin, 2010).

**FIGURE 1:**
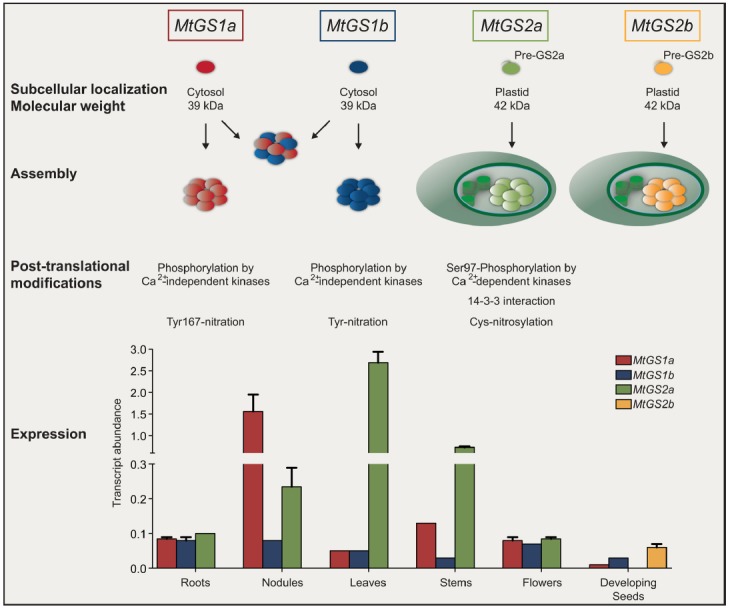
**Overview of glutamine synthetase expression and regulation in ***Medicago truncatula***.** GS is encoded by four expressed genes. *MtGS1a* and *MtGS1b* encode subunits of 39 kDa that can assemble homologously and heterologously in the cytosol. *MtGS2a* and *MtGS2b* encode precursor polypeptides of 47 kDa that upon import into the plastid are cleaved into mature polypeptides of 42 kDa. Independent of the subcellular localization, GS assembles into a decameric enzyme. Cytosolic and plastid isoenzymes are subjected to similar post-translational modifications but with mechanistic differences. Cytosolic GS proteins are phosphorylated by calcium–independent kinases whereas MtGS2a is phosphorylated by calcium-dependent kinases. The phosphorylation of MtGS2a at Serine 97 leads to 14-3-3 interaction and subsequent inactivation by proteolysis. The isoenzymes are also modified by nitric oxide, but cytosolic GS is inactivated by tyrosine nitration whereas the plastid located enzymes are inactivated by cysteine nitrosylation. The GS genes follow a differential pattern of expression in different organs of the plant (Seabra et al., 2013) where their encoded enzymes participate in different metabolic processes.

Owing the vital role and GS involvement in many aspects of the complex matrix of nitrogen metabolism, the enzyme must be tightly controlled. The first control occurs at the level of gene expression, an aspect that has been most intensively studied, but recent advances in molecular biology and protein biochemistry are revealing an impressive array of novel regulatory controls operating at many different levels to coordinate *in vivo* GS activity. It has been reported that GS is controlled at the level of mRNA processing and stability (Ortega et al., 2006; Stanulovic et al., 2006; Simon and Sengupta-Gopalan, 2010), translation initiation (Ortega et al., 2012) and at the post-translational level by phosphorylation and interaction with 14-3-3 (Moorhead et al., 1999; Finnemann and Schjoerring, 2000; Riedel et al., 2001; Lima et al., 2006a,b), interaction with other proteins (Masalkar et al., 2010; Seabra et al., 2013) protein turnover (Ortega et al., 1999, 2001) and by the nitric oxide (NO) induced post-translational modifications (PTMs) cysteine nitrosylation (Lindermayr et al., 2005; Melo et al., 2011) and tyrosine nitration (Cecconi et al., 2009; Lozano-Juste et al., 2011; Melo et al., 2011). Each isoenzyme appears to be regulated by a combination of mechanisms, coordinated to adapt the enzyme to operate at best performance under a specific metabolic context. Since GS isoenzymes cover a broad range of metabolic functions and the proteins share a high degree of amino-acid similarity, it is not simple to understand the specific metabolic function and the regulatory controls of individual members. This is especially difficult for the cytosolic enzymes, which are often simultaneously expressed in the same cell. Furthermore, the function of orthologous genes is not necessarily conserved in different plant species, and thus the function of a specific isoenzyme can hardly be inferred from one species to another. Recent studies covered the whole GS family in a single species, most of them on crop cereals due to the importance of GS for productivity (Swarbreck et al., 2011; Thomsen et al., 2014). In the case of legumes, an additional interest exists due to the strategic role that GS plays in the assimilation of ammonium produced by symbiotic nitrogen fixing rhizobia in root nodules. We have chosen the model legume *Medicago truncatula* to try to obtain a holistic view of GS regulation in a single legume species. This plant provides an excellent model system to study GS because it contains a simple GS gene family, with only four expressed genes, *MtGS1a* and *MtGS1b* encoding cytosolic polypeptides of 39 kDa, and *MtGS2a* and *MtGS2b*, encoding plastid-located polypeptides of 42 kDa, the latter of which is seed specific and unique to *M. truncatula* and closely related species (Seabra et al., 2010; Figure 1). The other three GS genes are expressed in almost all organs of the plant, but in a cell-specific manner. *MtGS2a* is highly expressed in all photosynthetic tissues, where it functions to assimilate the ammonia derived from photorespiration and nitrate reduction (Melo et al., 2003). The cytosolic *MtGS1a* is highly expressed in root nodules, where its main function is the assimilation the NH_4_^+^ produced by nitrogen fixation (Carvalho et al., 2000b), but it is also strongly expressed in the vascular bundles of all plant organs and probably involved in supplying glutamine for transport (Carvalho et al., 2000a). *MtGS1b* is ubiquitously expressed in all organs of the plant, functioning as a housekeeping gene, but its expression is increased during senescence and presumably it encodes the isoenzyme responsible for the reassimilation of the ammonia derived from protein catabolism (Carvalho et al., 2000a; Carvalho and Cullimore, 2003). Besides the discovery of a second plastid GS gene, our studies in *M. truncatula* revealed a number of new unsuspected mechanisms of GS regulation (Figure 1). This mini review summarizes exciting recent developments concerning the structure and regulation of GS isoenzymes, using the model legume *M. truncatula*.

## Dynamics of Enzyme Catalysis Revealed by the Structure Analysis of Cytosolic and Plastid-located GS

Determining the three-dimensional structures of closely related GS isoenzymes and their catalytic properties is critical to establish structure-function relationships. However, and in spite of the importance of GS in plants, the structure of the plant enzyme has only recently been elucidated (Unno et al., 2006; Torreira et al., 2014). For a long time it was believed that eukaryotic GS had a octameric architecture, based on extrapolation from the best-studied prokaryotic enzymes (Eisenberg et al., 1987). The determination of the atomic structure of the cytosolic GS1a from maize (ZmGS1a) and *M. truncatula* (MtGS1a) by X-ray crystallography revealed a decameric organization (Unno et al., 2006; Seabra et al., 2009; Torreira et al., 2014) that is common to the human and dog (Krajewski et al., 2008) and also to the yeast enzymes (He et al., 2009). Eukaryotic GS is arranged in two stacked (face-to-face) pentameric rings with extensive contacts established between subunits of the same ring and limited interactions with the subunits of the opposite ring. All monomers adopt a very similar conformation with each of the 10 active sites formed between two adjacent monomers from the same pentamer.

**FIGURE 2:**
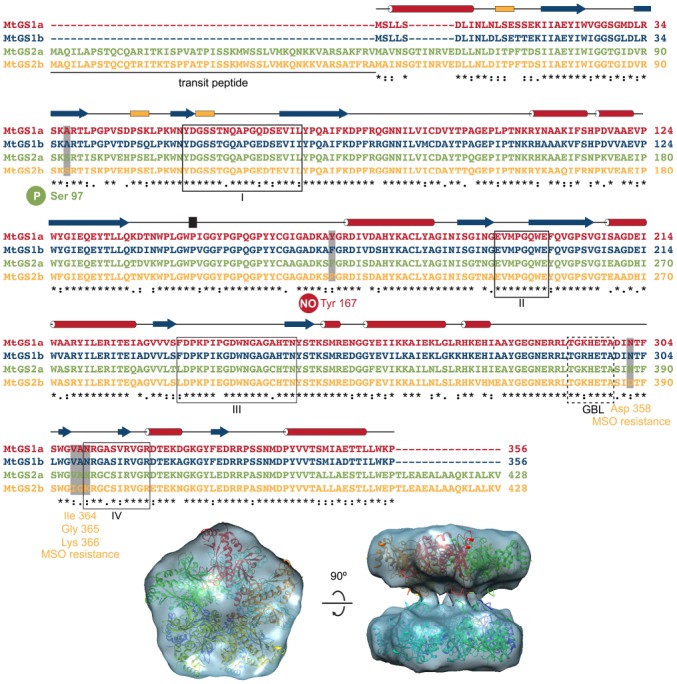
**Structural features of ***M. truncatula*** GSs.** The four *M. truncatula* GS polypeptides, MtGS1a, MtGS1b, MtGS2a, and MtGS2b share a high degree of amino-acid conservation. The secondary structure elements above the alignment, α-helices in red, 3_10_ helices in yellow and β-strands in blue correspond to the crystal structure of MtGS1a. Serine 97 is the regulatory phosphorylation site of MtGS2a (Lima et al., 2006a), and tyrosine 167 is the regulatory nitration site of MtGS1a (Melo et al., 2011). Boxed fragments I to IV represent the GS conserved regions involved in the active site, which were defined by Eisenberg et al. (1987). GBL corresponds to the conserved glutamate-binding loop referred in the text. The four MtGS2b amino-acid substitutions, presumably responsible for the resistance to the GS inhibitor methionine-S-sulfoximine (MSO), are also indicated. The bottom part of the figure shows a top and side view of the crystal structure of MtGS1a, in which each monomer is colored differently, fitting the electron cryo-microscopy reconstruction of MtGS2a (Torreira et al., 2014).

Here, we highlight some interesting features related to the dynamics of enzyme catalysis revealed by the structural analysis of the cytosolic (MtGS1a) and plastid-located GSs (MtGS2a) from *M. truncatula* (Torreira et al., 2014). The three-dimensional structure of unliganded MtGS1a was determined at 2.35 Ǻ resolution by X-ray crystallography (Figure 2; Seabra et al., 2009), and a comparison with the maize ortholog, whose structure was determined in complex with GS inhibitors (Unno et al., 2006) revealed some critical differences: The Trp141–Gly152 surface segment displays a different arrangement in the two enzymes, resulting in a 15° rotation in the relative position of the two rings in the free MtGS1a enzyme, when compared to the inhibited maize enzyme, suggesting that substrate binding and release could be related to the rotation of the pentameric rings (Torreira et al., 2014). The plastid-located MtGS2a is also a decamer composed of two superposed pentameric rings, as revealed by cryo-electron microscopy, but the association between the double-stacked rings appears to be more flexible than in MtGS1a (Torreira et al., 2014; Figure 2). The detection of two oligomeric states of MtGS2a by mass spectrometry under non-denaturing conditions, consistent with decamers and pentamers, argues in favor of the higher flexibility of inter-ring connections, suggesting a zipper-like mechanism for MtGS2a ring-ring assembly (Torreira et al., 2014). The funnel-shaped GS active site is structurally conserved between eukaryotic and the well-studied prokaryotic protein (Eisenberg et al., 2000), comprising the N-terminal β-grasp domain of one subunit and the highly curved β-sheet in the C-terminal catalytic domain of the neighboring subunit. Because MtGS1a was crystallized in the absence of substrates or inhibitors, the region that comprises the glutamate-binding loop (Thr293–Ala299) is disordered, leaving the entrance to the glutamate-binding site open at the narrower end of the funnel-shaped cavity. Interestingly, this flexibility of the glutamate-binding loop observed in the crystal structure of MtGS1a is predicted to be essential for the catalytic activity of the enzyme, allowing the exchange of newly synthesized glutamine for glutamate.

Understanding the three-dimensional structures of GS provides useful knowledge to develop new herbicides. Significant progress was achieved in *M. truncatula* with the determination of the catalytic properties of the four GS isoenzymes (Seabra et al., 2013) and their susceptibility to inhibition by phosphinothricin (PPT) and methionine-S-sulfoximine (MSO; Torreira et al., 2014). These are the active components of known herbicides, which have been shown to compete with glutamate binding. Interestingly, MtGS2b is extremely resistant to MSO inhibition. This significant functional difference likely arises from a few amino-acid changes that occur in the vicinity of the glutamate-binding site and are conserved in all other GS proteins (Asp358; Ile364-Gly365-Lys366 in MtGS2b and Asn302; Val308-Ala309-Asn310 in MtGS1a and other GS proteins; Figure 2). In its closed conformation, the glutamate-binding loop shields the catalytic intermediates (Liaw and Eisenberg, 1994) and the current available structural models indicate that these amino-acid modifications could stabilize the open conformation of the glutamate-binding loop, resulting in increased resistance to inhibition by MSO. These insights into the structural determinants of GS inhibition, open new possibilities to engineer GS for selective herbicide resistance in crop plants.

## GS Isoenzymes are Regulated by Multiple Post-translational Mechanisms

Post-translational modifications play an essential function in the regulation of metabolic pathways as they can, reversibly or irreversibly, alter the activity and function of enzymes, providing a short-term response to sudden metabolic fluctuations. GS proteins are known to suffer various PTMs, including phosphorylation, oxidative turnover, and nitration by NO. Yet, because of its complexity, a full understanding of the post-translational regulation of GS in plants remains elusive. Multiple isoenzymes, are often simultaneously expressed in the same organ, in some cases sharing similar size and isoelectric point, and a single GS isoenzyme can suffer more than one PTM. Thus, it is not easy to understand the effect of a specific PTM modification on individual GS proteins. *In vitro* studies using recombinant enzymes are useful to investigate the effect of a particular PTM on enzyme activity, but the physiological significance of PTMs requires studies *in planta*. *M. truncatula* is a useful model to study GS PTMs, because it contains only two cytosolic isoenzymes that can be separated by isoelectric focusing.

Our studies using recombinant enzymes from *M. truncatula* revealed that a single isoenzyme can suffer multiple PTMs, but the mechanisms of action and the effects on GS activity are isoenzyme specific. The picture is complex, with the three major *M. truncatula* GS isoenzymes MtGS1a, MtGS1b, and MtGS2a being regulated by phosphorylation (Lima et al., 2006a,b) and also by NO mediated PTMs, but by different mechanisms and with different outcomes on enzyme activity (Melo et al., 2011; Silva and Carvalho, 2013; Figure 1). The effect of NO is radical, leading to total enzyme inactivation, but with significant differences in the mechanisms of action for the three major GS proteins. The cytosolic enzymes MtGS1a and MtGS1b are irreversibly inactivated by tyrosine nitration, whereas MtGS2 appears to be reversibly inhibited by cysteine-nitrosylation. The intricacy of this regulation is further evidenced by the observation that the regulatory MtGS1a nitration residue, tyrosine 167 (Melo et al., 2011), is not conserved in MtGS1b (Figure 2), illustrating that in spite of the high amino-acid similarity between the two cytosolic isoenzymes, they are sufficiently different to be differentially affected by NO. The physiological significance of the differential NO-mediated PTMs in the different isoenzymes is still not fully understood, but the nodule enzyme MtGS1a has been shown to be nitrated *in vivo* and its nitration status was found to be related to higher NO content in the root nodules and reduced nitrogen fixation activity, leading to the proposal that the NO-mediated post-translational inactivation of GS is related to metabolite channeling to boost the antioxidant defenses (Melo et al., 2011; Silva and Carvalho, 2013).

Similarly, all three major *M. truncatula* isoenzymes are regulated by phosphorylation, but by different kinases and with different outcomes for GS activity. MtGS1a and MtGS1b are phosphorylated by calcium–independent kinase(s) (Lima et al., 2006b), whereas the plastid-located GS is phosphorylated by calcium-dependent kinase(s) (Lima et al., 2006a). Phosphorylation of GS2 at Serine 97 creates a 14-3-3 binding site and the complex is recognized by an unknown plant protease that cleaves the enzyme near the C-terminal causing enzyme inactivation (Lima et al., 2006a). 14-3-3 binding to phosphorylated GS has also been reported for GS2 in tobacco (Riedel et al., 2001) and GS1 in *Brassica napus* (Finnemann and Schjoerring, 2000), but with contrasting effects, as in these species 14-3-3s appears to act as GS activity activators, suggesting that either there are species-specific differences in the way GS is modulated or that senescence influences GS1 phosphorylation as the studies in *Brassica* used senescing leaves whereas the work in *M. truncatula* used mature leaves. The physiological effect of phosphorylation on the *M. truncatula* enzymes is still unclear, but cytosolic GS phosphorylation was found to be affected by light in leaves and by nitrogen fixation in root nodules, whereas GS2 phosphorylation was unaffected by these conditions (Lima et al., 2006b). Thus, it is clear that the enzymes are differentially phosphorylated under different physiological conditions, but understanding the physiological significance of phosphorylation will require further studies that must include quantitative measurements of phosphorylated versus non-phosphorylated GS.

There is also compelling evidence for the regulation of GS by protein–protein interactions in *M. truncatula*. The integration of GS proteins into organ-specific protein complexes with different molecular mass (Seabra et al., 2013) implies additional PTMs occurring under defined physiological conditions, denoting that there is still much to be discovered regarding the post-translational regulation of GS. The identification of the GS phosphorylating kinases, of the effect of phosphorylation on the activities of the enzymes and the characterization of proteins binding to each individual GS isoenzyme will be critical to understanding the molecular and physiological consequences of those PTMs for GS and for nitrogen metabolism.

## *Medicago truncatula* Contains a Second GS2 Gene, which is Seed-specific

Plants typically have a single gene encoding the plastid-located GS per haploid genome, *M. truncatula* is an exception to this rule as it contains an additional gene encoding GS2, which is expressed exclusively in the seeds. Seeds are essential elements for the dispersal of plant species and due to their high nutrient content are also important components of human diet. Seed yield is strongly dependent on efficient nitrogen remobilization from vegetative tissues, a process in which GS is determinant (Tabuchi et al., 2005; Martin et al., 2006). Nitrogen is remobilized mainly as asparagine and glutamine, which are subsequently deaminated in the developing seeds, implying that the resulting ammonia is re-assimilated by GS. Legume seeds are protein rich and it is predictable that GS activity is critical to achieve this high protein content. The existence of a seed-specific GS gene in *M. truncatula* (MtGS2b) with unique features, strongly suggests a specific role related to legume seed metabolism (Seabra et al., 2010).

The gene emerged from a recent duplication event estimated to have occurred 10 million years ago and hence after legume speciation. Thus, unlike the majority of plant GS gene families, *M. truncatula* and its closely-related species of the Vicioide clade have two genes encoding a plastid-located enzyme. Contrasting with the general expression of plastid-located GS in all organs of the plant, *MtGS2b* is exclusively expressed in developing seeds. Even thought both *M. truncatula* GS2 genes are transcribed during the initial stages of seed development, *MtGS2a* is repressed at the onset of seed filling, whereas *MtGS2b* is continuously expressed. This pattern of expression suggests that MtGS2b represents a functional adaptation to ammonium assimilation in legume seeds. Consistent with these observations are the unique kinetic properties of MtGS2b, which has the highest turnover rate and the lowest affinity for the three substrates, glutamate, ammonium and ATP of the four *M. truncatula* GS isoenzymes (Seabra et al., 2013). The low ammonium affinity of MtGS2b is in line with the predicted affinity of grain cytosolic GS isoforms (Goodall et al., 2013) pointing to the existence of special physiological conditions for ammonium assimilation in seeds.

In legumes, seed reserves are synthesized and stored in the embryo cotyledons rather than in the endosperm and it is conceivable that an increased plastid GS activity in embryos benefits a metabolism directed to the production of storage proteins. *M. truncatula*, as other legumes, have photoheterotrophic embryos, which ensures that the high energy required for the synthesis of storage compounds is fulfilled. High protein content also implies that resources are diverted from carbohydrate synthesis and channeled to amino-acid metabolism. By funneling glutamine into glutamate and aspartate production, GS plays a pivotal role in providing the building blocks for the biosynthesis of all amino-acids whose pathways are fully plastid-based (reviewed, in Wise and Hoober, 2007). It is also known that amino-acid metabolism contributes to the energy status of developing seeds by providing energy donors through the TCA cycle, a pathway also completely located in the plastid (reviewed, in Galili et al., 2014).

Given the important contribution of the metabolic pathways occurring inside the plastids of developing seeds to the synthesis of storage compounds, in particular to the synthesis of storage-proteins, we postulate that MtGS2b participates in the partitioning of assimilates into seed storage compounds. Plastid GS could also be involved in the remobilization of nitrogen released during the differentiation of embryonic chloroplasts into storage plastids. In such a role MtGS2b would be crucial to alleviate ammonium toxicity resulting from the degradation of the photosynthetic machinery, thereby affecting embryo development and consequently the whole plant life cycle.

## Concluding Remarks

This mini review emphasizes novel developments regarding the structure and regulation of GS isoenzymes, which were unveiled using the model legume *Medicago truncatula*. Major breakthroughs include the disclosure of the three-dimensional structure of the cytosolic and plastid-located enzymes which revealed a surprisingly dynamic molecule; the discovery of multiple PTMs such as phosphorylation, 14-3-3 interaction, tyrosine nitration and cysteine nitrosylation, which appear to be isoenzyme specific; and the existence of a second gene encoding a seed-specific GS2 with unique kinetic properties, which reflects a recent evolutionary functional specialization probably related to the production of seed storage proteins.

## Author Contributions

ARS and HC contributed equally to the content, drafting, and editing of the manuscript.

### Conflict of Interest Statement

The authors declare that the research was conducted in the absence of any commercial or financial relationships that could be construed as a potential conflict of interest.
